# Single-Nucleotide Polymorphism Array Technique Generating Valuable Risk-Stratification Information for Patients With Myelodysplastic Syndromes

**DOI:** 10.3389/fonc.2020.00962

**Published:** 2020-07-07

**Authors:** Xia Xiao, Xiaoyuan He, Qing Li, Wei Zhang, Haibo Zhu, Weihong Yang, Yuming Li, Li Geng, Hui Liu, Lijuan Li, Huaquan Wang, Rong Fu, Mingfeng Zhao, Zhong Chen, Zonghong Shao

**Affiliations:** ^1^Department of Hematology, Tianjin First Central Hospital, Tianjin, China; ^2^Department of Clinical Medicine, Nankai University School of Medicine, Tianjin, China; ^3^Department of Hematology, Tianjin Medical University General Hospital, Tianjin, China; ^4^Wuhan Kindstar Diagnostics Co./Kindstar Global Gene (Beijing) Technology, Inc., Wuhan, China

**Keywords:** myelodysplastic syndrome (MDS), idiopathic cytopenia of undetermined significance (ICUS), single-nucleotide polymorphism (SNP), chromosome aberrations, prognosis

## Abstract

**Background:** Chromosomal abnormalities play an important role in the diagnosis and prognosis of patients with myelodysplastic syndromes (MDSs). The single-nucleotide polymorphism array (SNP-A) technique has gained popularity due to its improved resolution compared to that of metaphase cytogenetic (MC) analysis.

**Methods:** A total of 376 individuals were recruited from two medical centers in China, including 350 patients and 26 healthy individuals. Among these patients, 200 were diagnosed with *de novo* MDS, 25 with myeloproliferative neoplasm (MPN), 63 with primary acute myeloid leukemia (AML), and 62 with idiopathic cytopenia of undetermined significance (ICUS). We evaluated the significance of abnormal chromosomes detected by SNP-A in the diagnosis and prognosis of MDS-related disorders.

**Results:** (1) When certain chromosomal abnormalities could not be detected by conventional MC methods, these abnormalities could be detected more efficiently by the SNP-A method. With SNP-A, the detection rates of submicroscopic or cryptic aberrations in the MDS, MPN, and AML patients with normal MC findings were 32.8, 30.8, and 30%, respectively. (2) The chromosomal abnormalities detected by SNP-A had a very important value for the prognosis of patients with MDSs, especially in the low-risk group. The survival of patients with abnormal chromosomes detected by SNP-A was significantly lower than that of patients with no detected chromosomal abnormalities; this difference was observed in overall survival (OS) (*P* = 0.001) and progression-free survival (PFS) [24 months vs. not reach (NR); *P* = 0.008]. The patients with multiple chromosomal abnormalities detected by SNP-A had an inferior prognosis, and SNP-A abnormalities (≥3 per patient) were found to be an independent predictor of poor prognosis in patients with MDSs [hazard ratio (HR) = 2.40, *P* = 0.002]. (3) Patients with ICUS may progress to myeloid malignancies, but most patients often maintain a stable ICUS status for many years without progression. An ICUS patient found to have an MDS-related karyotype would be rediagnosed with MDS. SNP-A can efficiently detect chromosomal abnormalities, which would be important for assessing the evolution of ICUS. In our study, 17 ICUS patients with SNP-A-detected abnormalities developed typical MDSs.

**Conclusions:** SNP-A can help evaluate the prognosis of patients with MDSs and better assess the risk of disease progression for patients with ICUS.

## Introduction

Myelodysplastic syndromes (MDSs) are a heterogeneous group of malignant hematopoietic disorders characterized by dysplastic changes in one or more cell lineages, ineffective hematopoiesis, and a variable predilection to the development of acute myeloid leukemia (AML) (1). Karyotype analysis provides useful diagnostic and prognostic information for many hematological malignancies. Some chromosomal lesions have a significant impact on the prognosis of MDS patients, and poor chromosomal lesions significantly affect the survival of patients (2–4). In the prognostic algorithm and the Revised International Prognostic Scoring System (IPSS-R) of MDSs, cytogenetic results account for an important proportion. In addition, recent studies have shown that MDS patients with certain cytogenetic abnormalities may benefit from targeted therapies (5, 6). However, the standard metaphase cytogenetic (MC) technique, in general, can only detect chromosomal rearrangements of more than 10 Mb in size. Furthermore, chromosome banding analysis is dependent on the cell proliferation of MDS clones in culture to obtain metaphases. Thus, the MC technique will miss many important chromosome abnormalities, resulting in genomic aberrations detectable in only 40–50% of MDS patients (7, 8). Notably, ~75–90% of chromosomal changes identified in MDSs are unbalanced aberrations, leading to gains or losses in all, or part, of specific chromosomes (3, 9, 10).

The single-nucleotide polymorphism array (SNP-A) technology relies on oligonucleotide probes corresponding to variants of the selected SNP allele. This method does not rely on cell division, has excellent resolution for unbalanced rearrangements, and overcomes some of the shortcomings of MC analysis. Since SNP-A has a higher analytical resolution than MC, SNP-A can detect submicroscopic or cryptic deletions or duplications. Another major advantage of SNP-A technology is its ability to recognize the loss of heterozygosity (LOH), which occurs when there is no simultaneous change in DNA copy number (CN), i.e., CN-neutral loss of heterozygosity. This defect is consistent with uniparental disomy (UPD). Acquired segmental UPD is increasingly recognized for its role in various tumors (11, 12). SNP-A-based genomic analysis has been applied in patients with various hematologic malignancies (2–4, 13, 14). A particularly interesting study by Mohamedali et al. (13) analyzed patients with low-risk MDS and found that 10% of these patients had a cryptic or submicroscopic deletion or duplication and 8% had gains. However, in general, the clinical significance of SNP-A-based analysis has not been fully realized.

The present study is aimed at developing a rational diagnostic algorithm for the detection of SNP-A-based genomic aberrations (unbalanced chromosome rearrangements and acquired UPDs) and establishing their clinical correlations in patients with MDS-related disorders. Based on the technical advantages of SNP-A, we assessed 376 cases of MDSs, various other myeloid disorders, and normal individuals. Our study represents the first such investigation in a large cohort of Chinese patients.

## Materials and Methods

### Patients

A total of 376 individuals were recruited from the Department of Hematology at Tianjin Medical University General Hospital and Tianjin First Central Hospital from April 2013 to September 2016. These individuals included 200 patients with *de novo* MDS, 25 with myeloproliferative neoplasm (MPN), 63 with primary AML, and 62 with idiopathic cytopenia of undetermined significance (ICUS) as well as 26 healthy individuals. The 62 ICUS patients were initially suspected of having MDS but were subsequently redefined as having ICUS due to lack of typical abnormal karyotypes and morphological dysplasia as well as a proportion of blast cells <5% (10, 15). The MPN and AML cases served as the positive controls, and the healthy individuals served as the normal controls for the purposes of assay validation (Table 1).

**Table 1:** Baseline characteristics of 376 cases in study.

**Characteristic**	***De novo* MDS**	**Pos ctl**		**NC**	**ICUS**
		**AML**	**MPN**		
Number	200	63	25	26	62
Age, years	12–87	11–91	50–87	26–74	9–74
Median	60	61	71	55	62
Male/Female	115/85	32/19	14/11	13/13	32/30
WBC, × 10^9^/L	0.4–38.2	0.2–265.9	2.3–24.5	4.3–9.5	1.2–11.5
Median	3.6	8.1	6.7	6.7	5.6
Hb, g/L	27–168	38–147	65–187	123–146	34–132
Median	82	89	102	132	66
PLT, × 10^9^/L	2–531	3–267	34–863	102–278	13–258
Median	96	42	167	176	71
Follow-up, months	6–42	8–39	6–40	–	6–42
Median	28	26	27	–	27

*MDS, myelodysplastic syndromes; AML, acute myeloid leukemia; MPN, myeloproliferative neoplasm; ICUS, idiopathic cytopenia of undetermined significance; WBC, white blood cell; Hb, hemoglobin; PLT, platelet; Pos ctl, positive control; NC, normal control*.

Clinical data used for the assessment included age, sex, blood cell counts, bone marrow morphology, blast counts, and survival times, including progression-free survival (PFS) and overall survival (OS), for all patients (Table 1). The diagnosis and classification of MDS were in accordance with the Vienna diagnosis standard and the 2008 WHO classification (10, 16). Among the 200 MDS patients, 115 were males and 85 were females, aged from 12 to 87 years old with a median age of 60 years. According to the 2008 WHO classification standard (17), 10 cases were classified as refractory anemia with ringed sideroblasts (RARS), 34 as refractory cytopenia with unilineage dysplasia (RCUD), 68 as refractory cytopenia with multilineage dysplasia (RCMD), 26 as refractory anemia with excess blasts-1 (RAEB-1), 46 as refractory anemia with excess blasts-2 (RAEB-2), nine as unclassified myelodysplastic syndrome (MDS-U), and seven as 5q-syndrome. In the prognostic evaluation of MDSs, IPSS-R was a commonly used method. IPSS-R was based on these characteristics (depth of cytopenias, splitting of marrow blasts <5%, and more precise cytogenetic subtypes). MDS patients were more precisely classified into all five IPSS-R categories, including Very low, Low, Intermediate, High, and Very high subgroups. Cytogenetic results accounted for an important proportion and could be divided into five categories, including Very good [–Y, del(11q)], Good [Normal, del(5q), del(12p), del(20q), double including del(5q)], Intermediate [del(7q), +8, +19, i(17q), any other single or double independent clones], Poor [−7, inv(3)/t(3q)/del(3q), double including −7/del(7q), complex: three abnormalities], and Very poor (complex: >3 abnormalities) subtypes (18). According to the IPSS-R standard, MDS patients in each subgroup were 10, 41, 54, 55, and 26, respectively; However, there were 14 cases not classified due to no cell growth available for MC analysis. The clinical features of these subgroups have been presented in Supplementary Table 1. The lower-risk group consisted of patients from the Very low, Low, and Intermediate categories of IPSS-R, and the higher-risk group was composed of patients from the High and Very high categories of IPSS-R. Patients were considered for clinical management driven by individual patient's clinical and biological characteristics and by physician preferences. Patients were managed according to the Chinese Expert Consensus on Diagnosis and Treatment of MDS (19). The goal of treatment for low-risk MDS patients was to improve the quality of life. The treatment was mainly supportive care, including blood transfusion, erythropoietin (EPO) and granulocyte colony-stimulating factor (G-CSF) administration, and removal of iron. Commonly used immunomodulation therapy drugs include thalidomide and lenalidomide. The target of MDS treatment in high-risk groups was to delay disease progression, prolong survival, and cure. The high-risk patients were treated with decitabine and/or chemotherapy. Hematopoietic stem cell transplantation was performed in eight of our patients.

All 376 recruited cases were subjected to SNP-A and MC studies on their BM samples. All samples were obtained at disease presentation.

This work was prospectively conducted in regard to specimen collection and clinical follow-up. OS was measured from day 0 to death from any cause (patients lost to follow-up were censored). PFS was defined as the time from day 0 to disease progression. This study was approved by the Ethics Committee of Tianjin Medical University General Hospital and Tianjin First Central Hospital. Patients and healthy controls gave their informed consent. The study was conducted in accordance with the Declaration of Helsinki.

### Cytogenetic Analysis

Cytogenetic analysis of bone marrow aspirates was performed according to standard methods. The chromosomal preparations were G-banded using trypsin and Giemsa (GTG), and the karyotypes were described according to the International System for Human Cytogenetic Nomenclature (ISCN) (20).

### Single-Nucleotide Polymorphism Array Analysis

SNP-A analysis was performed at Wuhan Kindstar Diagnostics Co./Kindstar Global gene (Beijing) Technology, Inc., P. R. China, by using the GeneChip Mapping 750K Assay Kit (CytoScan® 750K Assay Kit, Affymetrix, USA). Testing procedures were performed in strict accordance with the manufacturer's instructions and quality control standards, primarily including the steps of DNA extraction, enzyme digestion, connection, PCR, purification, fragmentation, labeling, hybridization, scanning, and data analysis. The detection instrument used was the GCS 3000Dx v.2 gene chip system, which is certified by the FDA/CE/CFDA, and the software used for data analysis was ChAS. The CytoScan 750K chip employed has more than 750,000 probes coated for the detection of genomic variance and covers 4,127 genes that include all the ISCA (International Standards for Cytogenomic Arrays) genes and 83% of the OMIM (Online Mendelian Inheritance in Man) disease-related genes. This chip can reliably detect copy number variations (CNVs), UPDs, and >10% of abnormal clones in mosaicism but is incapable of detecting balanced chromosome rearrangements and DNA point mutations. In the present study, three criteria were used to interpret a significant genomic aberration: First, the size of an identified aberration should be ≥400 Kb (for a gain), ≥400 Kb (for a loss), or ≥5 Mb (for a UPD) based on the manufacturer's recommendation and our own database. Second, the frequency of the identified aberration should be somewhat in concordance with the percentage of BM blasts in a patient, which could suggest that the aberration is likely acquired instead of constitutional in nature. Therefore, only aberrations in mosaic status (>10% of abnormal clones) were employed for further investigations. A threshold of 10% for mosaic identification was validated and provided by the manufacturer. Last, with regard to whether the aberration had been reported in association with respected disorders, related literature, and the Atlas of Genetics and Cytogenetics in Oncology and Hematology (http://atlasgeneticsoncology.org/Anomalies/Anomliste.html) should be reviewed and checked to identify possible disease relationships.

### Statistical Analysis

Categorical variables were compared using Fisher's exact test and the χ^2^ test. Variance analysis was used to compare measurement data. Survival analysis was performed using the Kaplan–Meier method, and the Cox proportional hazard model was used for univariate analysis and multivariate analysis. All *P-*values are two-tailed, and *P* < 0.05 indicates statistical significance. Statistical analyses were performed with SPSS version 19.0.

## Results

### Single-Nucleotide Polymorphism Array Analysis Led to a Higher Detection Rate of Chromosome Abnormalities

Our evaluation was performed on 376 cases that had been referred for identification of chromosome abnormalities by MC and SNP-A methods (Supplementary Table 2). MC allowed for the detection of 17 balanced rearrangements that were not detected by SNP-A. However, all the unbalanced chromosome aberrations identified by MC were also detected by SNP-A. In addition, SNP-A was able to detect many submicroscopic or cryptic chromosome abnormalities, which could not be detected by MC. The abnormality detection rate by SNP-A was 73.5, 72, and 69.8%, but by MC, it was 42, 48, and 36.5% in MDS, MPN, and AML patients, respectively. Comparing the two groups, the *P*-values were *P* ≤ 0.001, *P* = 0.148, and *P* ≤ 0.001, respectively. Notably, in our positive controls, the abnormal detection rates by both MC and SNP-A were higher in the MPN patients than in the AML patients likely due to the relatively small number of MPN patients enrolled in the study. Because our MPN and AML patients served as the positive controls, their detection results are only provided for assay validation purposes.

Importantly, in the 20 combined cases of MDS, MPN, and AML that had no informative MC findings (no cell growth available for MC analysis), 11 (55%) were found to be abnormal by SNP-A. In addition, with SNP-A analysis, the detection rates of submicroscopic or cryptic aberrations in the MDS, MPN, and AML patients with normal or no informative MC findings were 32.8, 30.8, and 30%, respectively. Furthermore, SNP-A-based aberrations in addition to the detection of MC in a patient were observed in 31% of the MDS, 50% of the MPN, and 30.4% of the AML patients. Notably, there were no abnormalities as detected by either MC or SNP-A in the normal controls.

Finally, even though all 62 ICUS patients were found to be normal by MC, 20 of them (32.2%) were identified as abnormal according to the SNP-A analysis.

### Single-Nucleotide Polymorphism Array Analysis Revealed More Complex Chromosome Abnormalities

**Figure 1:**
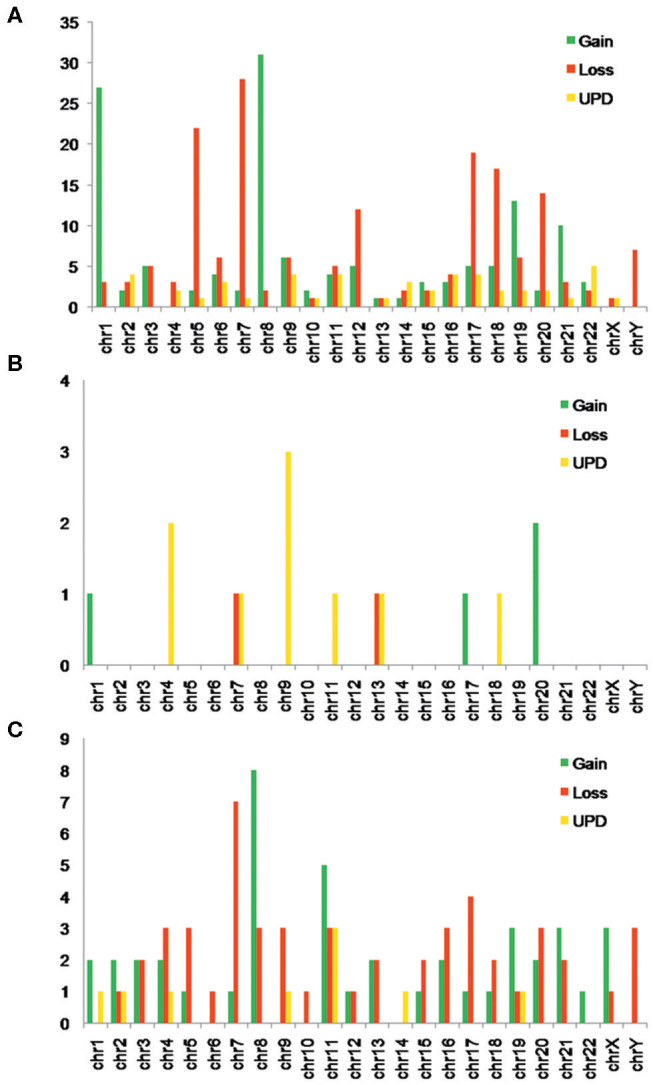
**(A)** Number of myelodysplastic syndrome (MDS) patients with different types of single-nucleotide polymorphism array (SNP-A) abnormalities in each chromosome. **(B)** Number of myeloproliferative neoplasm (MPN) patients with different types of SNP-A abnormalities in each chromosome. **(C)** Number of acute myeloid leukemia (AML) patients with different types of SNP-A abnormalities in each chromosome.

Using SNP-A, both CNVs and UPDs were observed in our MDS patients, with chromosome gains accounting for 42.0%, losses for 38.4%, and UPDs for 19.6%. The number of CNVs per patient ranged from 0 to 15, with a median number of 2.0 CNVs/patient. Notably, 88 of the 147 (59.9%) MDS patients with abnormal SNP-A detections showed 1–2 CNVs per patient, and 59 of the 147 (40.1%) showed ≥3 CNVs per patient. The SNP-A-detected abnormalities were found to involve essentially all 24 chromosomes, with chromosomes 1, 5, 7, 8, 9, 12, 17, 18, 19, 20, and 21 being affected relatively frequently. The detected chromosome aberrations by SNP-A mainly appeared as Gain 1q21, Loss 5q11, Loss 5q14, Loss5, Loss 7q11, Loss 7q22, Loss 7p21, Gain 8, Gain 9p13, Loss 9q21, UPD 9q21, Loss 12p11, Loss 12p13, Loss 17p11, Loss 17p13, Loss 18p11, Gain 19p13, Loss 19p13, Loss 20q11, and Loss 20q12. Notably, UPDs were observed to involve chromosomes 2, 4, 6, 9, 11, 19, and 22 (Figure 1A). All these findings were largely consistent with previously reported observations (2, 3, 5, 9).

In our positive controls (MPN and AML patients), many chromosomal abnormalities were also observed by SNP-A. Notably, these abnormalities were identified as commonly involving chromosomes 4, 7, 9, 13, and 20 in the MPN patients and chromosomes 7, 8, 11, and 17 in the AML patients (Figures 1B,C).

### Chromosomal Aberrations Detected by Single-Nucleotide Polymorphism Array Contributed to a Poor Prognosis in Patients With Myelodysplastic Syndromes

**Figure 2:**
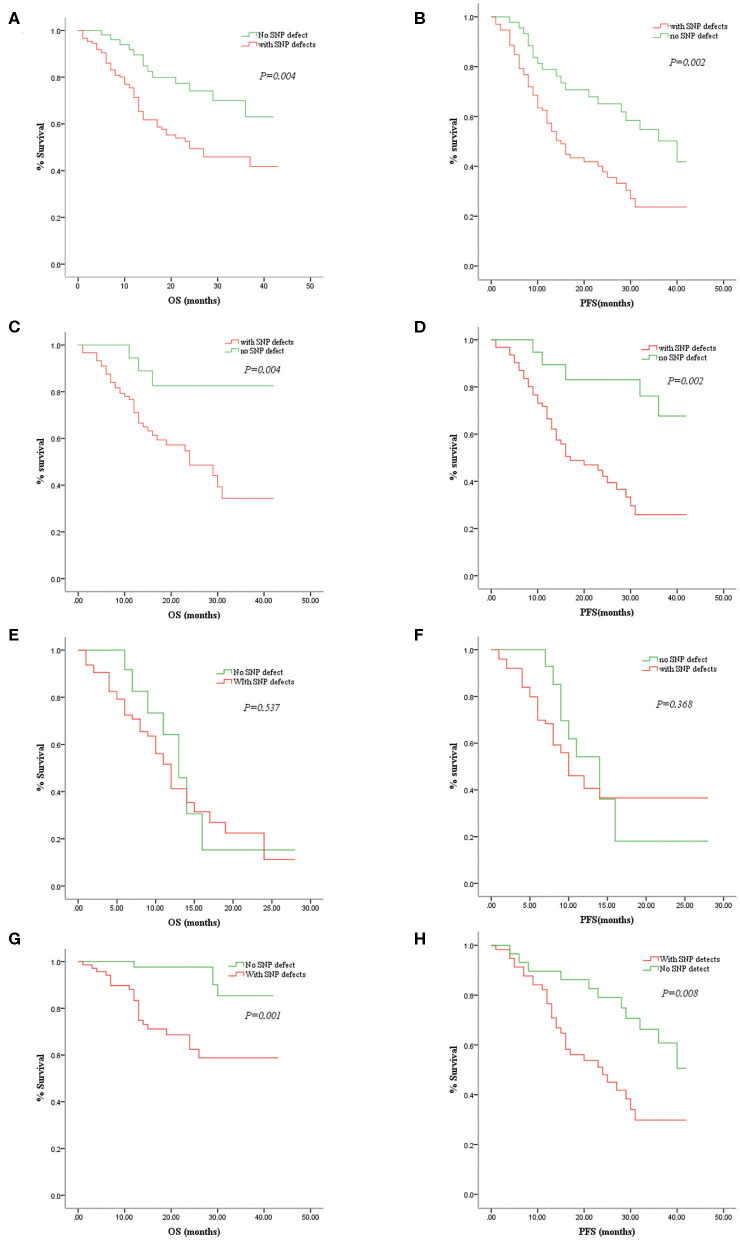
Correlations between overall survival (OS)/progression-free survival (PFS) and single-nucleotide polymorphism array (SNP-A) detections in patients with myelodysplastic syndrome (MDS). Comparison of the MDS patients with and without SNP-A aberrations in OS **(A)** and PFS **(B)**. Comparison of the MDS patients with abnormal SNP-A detections and without such additional SNP-A aberrations in OS **(C)** and PFS **(D)** of the normal or good cytogenetic findings by metaphase cytogenetics (MC). Comparison of the MDS patients with and without SNP-A aberrations in OS **(E)** and PFS **(F)** of the high-risk group. Comparison of the MDS patients with and without SNP-A aberrations in OS **(G)** and PFS **(H)** of the low-risk group.

IPSS-R evaluation predicts overall survival and leukemia-free survival of patients with primary MDSs (18). There is no doubt that cytogenetics is one of the most valuable indicators in assessing MDS prognosis in the “gold standard” scoring system. In our study, except for seven patients lost to follow-up, the remaining 193 patients with MDS were followed up for 6–42 months with a median time of 28 months. The MDS patients with SNP-A-detected abnormalities had significantly lower OS (24 months vs. NR; *P* = 0.004) and PFS (15 vs. 40 months; *P* = 0.002) than those without SNP-A abnormalities (Figures 2A,B). In addition, we evaluated the prognostic value of SNP-A analysis in MDS patients with normal karyotypes or good IPSS-R karyotypes by MC. Of these patients, the prognosis of the patients with abnormal SNP-A detections was significantly worse in terms of OS and PFS (Figures 2C,D).

According to the IPSS-R standard, high-risk and very-high-risk MDS patients were classified as the high-risk group, and very-low-risk, low-risk, and intermediate-risk MDS patients were classified as the low-risk group. In our study, SNP-A analysis did not demonstrate an advantage in prognostic assessment for the high-risk group (Figures 2E,F). However, in the low-risk group, the patients with abnormal SNP-A detections had a significantly shorter survival time than patients without SNP-A aberrations (Figures 2G,H). Therefore, for MDS patients with a low-risk evaluation according to IPSS-R, SNP-A analysis seems to have a more significant impact on prognostic prediction.

Finally, in one patient, the number of SNP-A abnormalities, clinical features (including sex, age, blood counts, bone marrow blasts), and MC findings were also used to evaluate the prognosis of MDS patients by multivariable analysis (Table 2). The number of SNP-A abnormalities (≥3 per patient) was an independent predictor of poor prognosis in the patients with MDS [hazard ratio (HR) = 2.40, *P* = 0.002]. Our investigations provided valuable additional risk-stratification information to the standard IPSS-R scoring system.

**Table 2:** Multivariable analysis of clinical data, MC findings, and number of SNP-A aberrations.

**Factor**	**Hazard ratio (95% CI)**	***P***
Age	1.73 (0.75–4.07)	0.002
Sex (male vs. female)	1.47 (1.01–1.69)	0.007
NEU (×10^9^/L) (<0.8 vs. ≥0.8)	1.19 (0.81–2.92)	0.029
Hb (g/L) (<80 vs. 80–100 vs. ≥100)	1.52 (1.06–4.02)	0.016
Plt (×10^9^/L) (<50 vs. 50–100 vs. ≥100)	1.06 (0.58–1.52)	0.030
BM blasts (%) (<5 vs. 5–10 vs. >10)	1.79 (1.04–3.47)	0.016
MC (very good, good, intermediate vs. poor, very poor)	2.22 (0.79–6.12)	0.008
Number of SNP-A aberrations (≥3 vs. <3)	2.40 (1.48–9.57)	0.002

*NEU, neutrophil; Hb, hemoglobin; Plt, platelet; BM, bone marrow; MC, metaphase cytogenetics; SNP-A, single nucleotide polymorphism array*.

### Chromosomal Aberrations Detected by Single-Nucleotide Polymorphism Array Were Closely Associated With a High Risk of Transformation to Typical Myelodysplastic Syndrome in Patients With Idiopathic Cytopenia of Undetermined Significance

Patients with ICUS may progress to myeloid malignancies, but most patients often maintain a stable ICUS status for many years without progression. An ICUS patient once identified as having an abnormal karyotype that meets the MDS criteria would be rediagnosed with MDS. SNP-A can efficiently detect chromosomal abnormalities, which is important for assessing the evolution of the disease. In our study, 20 of the 62 ICUS patients were found to have chromosomal abnormalities by SNP-A technology. These abnormalities affected almost all chromosomes except chromosomes 2, 10, 11, 13, 16, and X (Table 3). These 20 ICUS patients with SNP-A aberrations were followed up for a median of 11 months (6–20 months). Notably, 17 of them (85%) transformed to typical MDS, and the remaining three (15%) transformed to aplastic anemia (AA) (Table 3). However, the other 42 ICUS patients without SNP-A abnormalities were also followed up for a median of 12 months (3–24 months), and none of them were converted to MDS. Therefore, chromosomal abnormalities detected by SNP-A were closely associated with a high risk of disease transformation in patients with ICUS.

**Table 3:** Aberrations detected by SNP-A in 20 ICUS patients.

**Patients**	**Aberrations**	Diagnosis^*^	Time^**^
1	UPD (17q11.1-q11.2)	RAEB-1	8 months
2	Loss(20q), Gain(21q), UPD(14q)	MDS-U	12 months
3	Loss(5q21.1-qter), Loss(12p), Loss(17q)	RCMD	6 months
4	Loss(Y)	AA	13 months
5	UPD(19p)	AA	8 months
6	UPD(6p)	RCUD	10 months
7	Gain(8)	RCUD	12 months
8	UPD(14q)	RCMD	10 months
9	Gain(1q)	RCMD	15 months
10	Loss(20q)	RCUD	13 months
11	Loss(3p), Gain(18q), UPD(9p,12q)	RCMD	6 months
12	UPD(19q)	RCUD	14 months
13	Gain(1q), Loss(7q), UPD(15q,17q)	RCMD	8 months
14	Gain(8)	MDS-U	10 months
15	Loss(Y)	RCUD	14 months
16	UPD(4q)	RCMD	18 months
17	Gain(8)	AA	9 months
18	UPD(4q)	RCUD	17 months
19	Loss(4q,5q,11p,17), Gain(21q)	RAEB-1	7 months
20	UPD(5q)	RCUD	20 months

*UPD, uniparental disomy; RAEB-1, refractory anemia with excess blasts-1; MDS-U, myelodysplastic syndrome, unclassified; RCMD, refractory cytopenia with multilineage dysplasia; RCUD, refractory cytopenia with unilineage dysplasia; AA, aplastic anemia*.

* *Diagnosis after transformation from ICUS*.

** *Follow-up time from initial diagnosis to disease transformation*.

## Discussion

The global profiling of DNA copy number changes in cancer cells through the use of microarray platforms is extremely attractive because it provides an unparalleled opportunity to uncover elusive genomic aberrations that are critical to tumorigenesis and progression. SNP-A technology allows for the capture of DNA copy number changes and SNP-based genotypes at sub base resolution, which helps detect small-scale genomic lesions and UPDs. A series of SNP-A-based studies have been performed on hematologic disorders, including acute lymphoblastic leukemia (21), MDS (22–25), myeloma (26), leukemias (27–29), and lymphomas (30).

From a technological point of view, our investigations have demonstrated that the detection of chromosomal abnormalities can be improved significantly by using the SNP-A technique for patients with MDS. From the following several aspects of data analyses, even somewhat confirmatory for previous findings in nature, we could still better appreciate the technical advantages of SNP-A over MC in detecting chromosomal aberrations. First, in our study, the abnormal detection rate by SNP-A for the patients with MDS and for the positive controls (MPN and AML patients) was higher than that obtained by MC. Second, SNP-A allowed for the detection of cryptic chromosomal lesions in the MDS patients and the positive controls with normal, abnormal, or even no informative MC findings, meaningfully demonstrating the technical reliability of SNP-A analysis. Third, SNP-A can detect chromosome deletions, gains, and UPDs. Acquired UPDs have been described in several malignancies (31–33), but due to the inability of MC to identify them, UPDs have remained largely elusive in many hematological disorders. Acquired segmental UPD is likely the result of mitotic recombination and appears to be a common event in MDS (24, 34, 35). In our study, acquired UPDs were observed in 19.6% of the MDS patients, with chromosomes 2, 4, 6, 9, 11, 19, and 22 being involved, which is largely consistent with previous reports. Finally, from a practical point of view, we would still recommend the combined application of MC and SNP-A for detection because MC can offset the inability of SNP-A to identify balanced chromosome rearrangements.

From a clinical point of view, our studies offered the following findings either not previously reported or less emphasized:

(1) Remarkably, in our study, 20 of the 62 ICUS patients had abnormal SNP-A detections, and 17 of these 20 patients progressed to typical MDSs with a progression time of 6–20 months and a median progression time of 11 months. Thus, abnormal SNP-A detections may predict the transformation to MDSs in advance for patients with ICUS, which would lead to disease monitoring and early intervention.

(2) It is likely that the presence of chromosome abnormalities as detected by SNP-A is responsible for the prediction of clinical phenotype and prognosis. A series of studies have shown that SNP-A detection is closely associated with prognosis (24–26). In this regard, our current study further strengthened the clinical value of SNP-A detection in prognostic assessment for patients with MDS. As a result, the patients with a normal SNP-A finding likely had a more favorable prognosis; SNP-A detection had an especially important value for prognostic assessment of the MDS patients in the low-risk group; the number of abnormalities (≥3 per patient) was observed to be an independent predictor of poor prognosis. Therefore, our observations are of significant clinical value and provide additional information important for further risk-stratification assessment of patients with MDSs. Based on our findings and those of previous reports, it is now evident that a combination of MC and SNP-A methods would provide a more precise assessment of the prognosis of patients with MDSs. Recently, a series of studies (2, 6, 22, 36) showed that total genomic alterations detected by SNP-A were predictive of overall survival in a cohort of patients with MDSs or other related hematological disorders who received demethylation-based treatment, which certainly deserves further investigation.

A better understanding of the strength and weakness of each technique in a clinical setting is of extreme importance. SNP-A can detect loss of heterozygosity and serve as a useful complement to MC by capturing additional submicroscopic or cryptic chromosome gains or deletions. However, SNP-A can only detect chromosomal or chromosome-fragment-size aberrations but cannot detect single gene-based mutations. Recently, Choi et al. (37) used a more sensitive SNP-A approach (Affymetrix CytoScan HD) to investigate submicroscopic or cryptic chromosome aberrations in MDS patients. This CytoScan HD platform had ~2.7 million coated probes (much more than that of the CytoScan 750K chip employed in our study) and was able to detect gains or losses of more than 35 markers within or including a known clinically significant cancer-related gene. Thus, in the study by Choi et al., they could identify much smaller cryptic abnormalities, such as KMT2A partial tandem duplication and deletion involving the TET2 gene, that are often smaller than 100 kb in size. Certainly, the CytoScan 750K-based SNP-A platform adopted in our study cannot reach such a greater sensitivity in detection. Based on the detection of chromosome-fragment sized aberrations (often >400 kb in size), our study provided several findings either not previously reported or less emphasized as described above and should be considered valuable information complementary to Choi's findings. Next-generation sequencing (NGS) focuses more on gene mutation analysis. Mutant genes can be detected in more than 80% of MDS patients, and most mutations are not specific and usually have uncertain significance (38). Although NGS makes it increasingly easy to detect fusions and mutations, not all cytogenetic abnormalities can be detected by NGS. Therefore, if feasible, these techniques should be combined to contribute to the study of genomic aberrations for better and more precise management of patients with MDS (39–42).

## Data Availability Statement

The datasets generated for this study are available on request to the corresponding author.

## Ethics Statement

This study was approved by the Ethic committee of Tianjin Medical University General Hospital and Tianjin First Central Hospital. Patients and healthy controls gave their informed consent. The study was conducted in accordance with the Declaration of Helsinki.

## Author's Note

Presented in abstract form at the 59th annual meeting of the American Society of Hematology, Atlanta, GA, December 9, 2017. TITLE: Chromosome aberrations detected by SNP array technique indicating a high risk of MDS transformation in patients with ICUS and poor prognosis of patients with MDS.

## Author Contributions

MZ, ZC, and ZS designed the study. XX and XH collected and analyzed the data and wrote the manuscript. QL, WZ, HZ, WY, YL, LG, HL, LL, HW, and RF provided clinical data. MZ, ZC, and ZS reviewed the manuscript and contributed to the final draft. All authors contributed to the article and approved the submitted version.

## Conflict of Interest

WY and ZC were employed by Wuhan Kindstar Diagnostics Co./Kindstar Global gene (Beijing) Technology, Inc. The remaining authors declare that the research was conducted in the absence of any commercial or financial relationships that could be construed as a potential conflict of interest.
