# Supplementary material for: Single-Nucleotide Polymorphism Array Technique Generating Valuable Risk-Stratification Information for Patients With Myelodysplastic Syndromes
Source: Front Oncol. 2020 Jul 7;10:962. doi: 10.3389/fonc.2020.00962 (PMC7358551; doi:10.3389/fonc.2020.00962)
Supplement: Supplementary file 1 [file Table_1.docx]

Supplementary table 1. The clinical features of patients in each IPSS-R subgroup

| Clinical features | Very low | Low | Intermediate | High | Very high |
| --- | --- | --- | --- | --- | --- |
| Number | 10 | 41 | 54 | 55 | 26 |
| Age, years | 35-64 | 21-81 | 29-87 | 20-84 | 12-83 |
| Median | 55 | 60 | 59 | 56 | 62 |
| WBC, ×10^9^/L | 3.7-14.1 | 1.6-14.5 | 1.2-38.2 | 0.4-25.7 | 1.0-9.9 |
| Median | 6.7 | 3.7 | 3.7 | 3.4 | 2.4 |
| Hb, g/L | 84-168 | 40-134 | 39-164 | 38-144 | 27-137 |
| Median | 103 | 82 | 73 | 82 | 79 |
| PLT, ×10^9^/L | 16-133 | 2-531 | 7-367 | 6-516 | 2-247 |
| Median | 87 | 155 | 91 | 57 | 35 |
